# Editorial: Emerging Therapies for Malignant Mesothelioma

**DOI:** 10.3389/fonc.2020.00939

**Published:** 2020-06-12

**Authors:** Nico van Zandwijk, Glen Reid, Paul Baas

**Affiliations:** ^1^Concord Repatriation General Hospital, Sydney Local Health District/University of Sydney, Concord, NSW, Australia; ^2^Department of Pathology, Dunedin School of Medicine, University of Otago, Dunedin, New Zealand; ^3^Department of Thoracic Oncology, Netherland Cancer Institute, Amsterdam, Netherlands

**Keywords:** malignant mesothelioma, tumor biology, preclinical models, immune environment, angiogenesis inhibitors, chemotherapy, immunotherapy, microRNA

Malignant mesothelioma, resistant to most currently available therapies, is associated with the lowest survival rates of any major cancer type. Despite intensive efforts, significant improvements in patient outcomes have remained out of reach. Although some activity of experimental approaches such as intrapleural pro-inflammatory cytokines was noted in the 1990s (1–4), chemotherapy-based therapies, surgery and radiotherapy dominated clinical research into mesothelioma treatment through the 1990s and 2000s. This was particularly the case after the pemetrexed/cisplatin combination was established as the backbone of systemic therapy for malignant pleural mesothelioma (MPM) in 2003 (5). Although radical surgery continues to be associated with superior survival figures, it is unable to shift survival beyond the 2-year mark (6) and the reality is that < 10% of patients will be judged eligible for radical multimodality therapy. Moreover, the peri-operative mortality of extra-pleural pneumonectomy turned out to be considerable, eliciting discussions about acceptable levels of surgical morbidity/mortality and the feasibility of aggressive multimodality approaches (7–9).

It has taken many years for mesothelioma research to take a different direction, and this has largely followed advances in the treatment of other cancer types. However, despite the promise of these new approaches, failures have outnumbered successes. In stark contrast to the beneficial effects of targeted therapy in non-small cell lung cancer and other cancers driven by mutated oncogenes, targeted therapy approaches were largely unsuccessful in MPM. Despite frequent overexpression of EGFR in MPM, TKIs, and antibodies blocking the receptor lacked sufficient clinical activity. The addition of bevacizumab to pemetrexed/cisplatin led to a significant survival advantage, this gain was only a modest 3 months (10). In retrospect, these observations should not have surprised us, considering the relatively low mutational burden in mesothelioma and relative lack of oncogenic drivers (11, 12).

After little improvement in patient outcomes despite the intensive efforts of the past two decades, the recent advances using novel clinical and experimental approaches for MPM provide new hope. The rapid changes in prognosis of melanoma and non-small cell lung cancer as a consequence of treatment with immune-checkpoint inhibitors have now found their way into the mesothelioma field (13). As a consequence of some positive studies in the second-line setting, the National Comprehensive Cancer Network (NCCN) guidelines have recently accepted pembrolizumab and nivolumab with or without ipilimumab as salvage therapy (NCCN guidelines Version 2.2019-April 1, 2019). At the same time the mesothelioma community is also paying attention to other immunotherapy approaches, such as tumor vaccines, immunotoxins, and targeted T-cells. Additional experimental approaches including microRNA replacement therapy, have also shown signs of clinical efficacy (14).

Therefore, it is appropriate to review recent translational research studies and the early clinical experience with novel treatment approaches for mesothelioma. Thirty-five mesothelioma researchers from around the world have made a contribution, and it is a great privilege for the editors to introduce this series of 10 articles which summarize our increasing insight into mesothelioma biology and the gradual change in treatment approaches for MPM.

Our article collection begins with pre-clinical lab studies before discussing new clinic approaches. Testa and Berns are the first to review rodent models that have greatly assisted in increasing our understanding of the pathophysiology of mesothelioma. Blanquart et al. have a similar goal and discuss the pros and cons of the different preclinical mesothelioma models used, including organoids. In an opinion paper, Felley-Bosco and Gray concentrate on tumor suppressor genes, ferroptosis, and resistance of mesothelial cells against apoptosis. Chu et al. seek explanations for the mixed results of immunotherapy trials by reviewing the tumor micro-environment of mesothelioma, and Reid et al. highlight the potential of restoring levels of tumor-suppressive microRNAs in MPM in the lab and clinic.

The strong rationale behind the inhibition of angiogenesis in a highly inflammatory tumor such as mesothelioma is detailed by Nowak et al. while de Gooijer et al. provide an overview of the rapidly expanding clinical experience with immune checkpoints inhibitors in MPM. The promise of cellular immunotherapy in MPM is given by Belderbos et al.. Finally, the last two decades of clinical trials in MPM are comprehensively reviewed by two separate groups (Cantini et al.; Nicolini et al.). Both reviews underline the importance of well-designed clinical trials to improve treatment outcomes in MPM and to incorporate biomarkers validated in the translational setting. Considering past experience, it is very unlikely that we will discover a one-size-fits-all therapy for MPM patients. However, with the spectacular increase in translational mesothelioma data witnessed in the last decade, there is hope that this will eventually translate into better treatment outcomes for patients affected by one of the most recalcitrant solid tumors.

## Author Contributions

NZ, GR, and PB contributed to the writing and reviewing of this editorial.

## Conflict of Interest

The authors declare that the research was conducted in the absence of any commercial or financial relationships that could be construed as a potential conflict of interest.
